# The metrics and correlates of physician migration from Africa

**DOI:** 10.1186/1471-2458-7-83

**Published:** 2007-05-17

**Authors:** Onyebuchi A Arah

**Affiliations:** 1 Department of Social Medicine, Academic Medical Center, University of Amsterdam, PO Box 22700, Amsterdam 1100 DE, The Netherlands

## Abstract

**Background:**

Physician migration from poor to rich countries is considered an important contributor to the growing health workforce crisis in the developing world. This is particularly true for Africa. The perceived magnitude of such migration for each source country might, however, depend on the choice of metrics used in the analysis. This study examined the influence of choice of migration metrics on the rankings of African countries that suffered the most physician migration, and investigated the correlates of physician migration.

**Methods:**

Ranking and correlational analyses were conducted on African physician migration data adjusted for bilateral net flows, and supplemented with developmental, economic and health system data. The setting was the 53 African birth countries of African-born physicians working in nine wealthier destination countries. Three metrics of physician migration were used: total number of physician émigrés; emigration fraction defined as the proportion of the potential physician pool working in destination countries; and physician migration density defined as the number of physician émigrés per 1000 population of the African source country.

**Results:**

Rankings based on any of the migration metrics differed substantially from those based on the other two metrics. Although the emigration fraction and physician migration density metrics gave proportionality to the migration crisis, only the latter was consistently associated with source countries' workforce capacity, health, health spending, economic and development characteristics. As such, higher physician migration density was seen among African countries with relatively higher health workforce capacity (0.401 ≤ *r *≤ 0.694, *p *≤ 0.011), health status, health spending, and development.

**Conclusion:**

The perceived magnitude of physician migration is sensitive to the choice of metrics. Complementing the emigration fraction, the physician migration density is a metric which gives a different but proportionate picture of which African countries stand to lose relatively more of its physicians with unchecked migration. The nature of health policies geared at health-worker migration can be expected to depend on the choice of migration metrics.

## Background

The World Health Organization estimates that some 57 countries, many in Africa, face crippling health workforce shortages and that the global deficits of physicians, nurses and midwives easily exceed 2.4 million [1]. An often cited major contributor to the workforce shortages in Africa is migration of physicians and nurses to rich western countries, especially to the Anglophone health systems of United States (US), Canada, Australia and the United Kingdom (UK) [1-6], but also to other member states of the Organization for Economic Cooperation and Development (OECD) [7,8]. This is particularly worrisome given the insufficient supply of health-workers in these African countries or the so-called *source countries*: they have only 3% of the world's health-workers although they represent 11% of the global population and endure 24% of the global burden of disease [1,9,10]. The migration is seen as so unjust (given its from-poor-to-rich flow) and threatening to already weak systems that there are even calls for migration reversal and immediate cessation of active recruitment from such deprived health systems [11,12].

To quantify the magnitude of migration many publications have usually relied on two metrics: absolute numbers of émigrés and the proportion of the source country's health workforce that has migrated – the emigration rate or fraction. For instance, Ghana and South Africa are said to have lost 1,639 and 7,363 physicians (that is, medical doctors) respectively to at least eight more developed *destination countries *including the UK and the US, but are estimated to have emigration fractions of 56% and 21% respectively [13]. Based on the number of physician émigrés, Ghana's loss is less than that of South Africa, but given her emigration fraction, Ghana has lost more physicians. These two metrics are not the only possible migration metrics, and the differences in the magnitude of physician migration given by the different metrics point to the likelihood that different metrics paint different perspectives of the migration problem. The choice of metrics is particularly important for understanding health-worker migration patterns and correlates at the macro-level.

Given the paucity of research into this issue, I studied the effect of choice of migration metrics on quantifying the extent and correlates of physician migration from Africa to the major destinations in North America, Europe, Australia and South Africa. The study was particularly concerned with how African countries ranked when different migration metrics were used and how such metrics correlated with the source countries' profiles.

## Methods

A new database – the first of its kind – on cumulative bilateral net migration of health professionals from 53 African countries to nine wealthier destinations mostly in North America and Europe was used to extract data on physician migration [13]. The full list of the source countries is given in Additional File 1 online. Eight of the nine destination countries, which also hosted more than 94% of all African-born university trained residents in the Organisation for Economic Cooperation and Development (OECD) countries [7], were: the United Kingdom (UK), the United States (USA), France, Canada, Australia, Belgium, Portugal, and Spain [13]. South Africa was included as the ninth destination country given that it is the most important non-OECD country to which other African physicians migrate [13]. The database defines an African physician as one born in Africa and currently employed as a medical doctor, thus effectively excluding émigrés who no longer practice medicine [13]. Other relevant data on the health workforce were obtained from the World Health Organization (WHO) [1,14,15] and the Joint Learning Initiative (JLI) [4] sources, often based on *circa *year 2000 data (range of data availability: 1993–2004). I also used WHO [16], JLI [4] and World Bank [17] databases to extract data on health status and health system spending. Economic and social development data were triangulated and taken from JLI [4], World Bank [17] and United Nations (UN) [18] sources. The details of extracted variables, their definitions, sources and year of data are given in Additional File 2.

To quantify the extent of physician migration from Africa to the nine destinations, I used three metrics: total number of physician émigrés, emigration fraction, and physician migration density. The number of physician émigrés refers to the total number of currently employed doctors who were born in Africa and have lived long enough in the destination country to be part of that country's census [13]. The emigration fraction is a well-known migration metric which is the ratio of the number of physician émigrés to the sum of the number of physicians remaining at home in Africa and the number of émigrés [2,3,13]. The physician migration density is a recently proposed metric based on the number of physician émigrés per 1000 population of each African country [19-21].

Both the emigration fraction and physician migration density give some proportionality. The former related to the physician pool of a source country (thus allowing us to say something about the proportionate effect of migration on the size of the workforce) while the migration density metric was weighted by the source country's population [20]. This study hypothesized that each migration metric would give a different picture of which source countries suffer relatively more physician emigration because each metric represented a different notion.

To relate each migration metric to the source countries' characteristics, I checked for migration patterns using traditionally available and commonly used data on health workforce capacity, health status, health system spending and economic and social development profiles of the source countries. Health workforce variables included remaining or current physician, nurse, and medical school densities. Health status was measured as infant mortality and under-five mortality rates, and healthy life expectancy at birth. Total health spending and the share of that spending (in the form of official development aid for health) from external resources were both used to capture health system spending. Finally, I operationalized economic and social development as gross national income per capita, poverty (proportion living on less international $1-per-day), female literacy (percentage of females aged 15 years or older who are literate), and the Human Development Index (a composite variable reflecting a country's human development attainment in terms of health, knowledge and standard of living) [20]. Additional File 2 online details all these variables.

Based on each of the three metrics I constructed rankings of African countries with respect to their physician migration to the UK, USA, France and Canada – the top four destinations – and for all nine destinations combined. I also estimated Pearson's correlations between these migration metrics and national characteristics of the African countries. Scatter plots were also used to examine the correlations between the metrics and source countries' characteristics. All variables were log-transformed, and analyses were conducted in SPSS version 12.0.2 (SPSS Inc, Chicago, IL, 2003) and Microsoft^® ^Office Excel 2003 SP2 (Microsoft^®^, Redmond, WA, 2003).

## Results

Table 1 shows the rankings of the top five African countries with the most physician migration to the UK, USA, France, and Canada, and to all nine destinations combined. No country retained the same rank with all three different migration metrics. In most situations, an entirely different set of countries replaced the top five or the bottom five source countries when the migration metric was changed. For instance, while South Africa lost the most physicians (3,509) in absolute terms to the UK, Malawi lost the highest proportion of its physicians (38.4%) to the UK, and Seychelles had the highest physician migration density (0.36 doctors per 1000 Seychelles population) with regards to the UK. Algeria had the most émigrés (13,639) combined in all nine destinations but was neither in the top five nor the top ten of countries with the highest emigration fractions although it ranked number five in terms of physician migration density. Furthermore, Mozambique, which had the highest emigration fraction with respect to all nine destinations, only ranked 21^st ^out of 53 on the physician migration density. Additional File 3 gives the top ten and bottom ten African countries per migration metric.

**Table 1:** Top five African birth countries of foreign physicians registered in different destination countries, ranked by number of physician émigrés, emigration fraction, and physician migration density (in descending order)

**Destination countries**	**Source Countries**		
	**Total number of physician émigrés**^a^	**Emigration fraction**^b^	**Physician migration density**^c^

**UK**			
	South Africa	Malawi	Seychelles
	Kenya	Kenya	Mauritius
	Nigeria	Zambia	Kenya
	Egypt	Tanzania	South Africa
	Uganda	Uganda	Libya
			
**USA**			
	Egypt	Liberia	Egypt
	Nigeria	Gambia	South Africa
	South Africa	Ghana	Ghana
	Kenya	Ethiopia	Liberia
	Ghana	Eritrea	Cape Verde
			
**France**			
	Algeria	Senegal	Algeria
	Morocco	Algeria	Tunisia
	Tunisia	Central African Republic	Mauritius
	Madagascar	Togo	Morocco
	Senegal	Madagascar	Senegal
			
**Canada**			
	South Africa	Tanzania	Seychelles
	Egypt	Eritrea	Mauritius
	Tanzania	Mauritius	South Africa
	Kenya	Seychelles	Namibia
	Uganda	Somalia	Libya
			
**All nine destination countries^#^**			
	Algeria	Mozambique	Mauritius
	South Africa	Guinea-Bissau	São Tomé & Principe
	Egypt	Angola	Seychelles
	Morocco	Liberia	Cape Verde
	Nigeria	Equatorial Guinea	Algeria

^#^United Kingdom, United States, France, Canada, Australia, Belgium, Portugal, Spain, and South Africa

^a^Total number of physician émigrés: the total number of currently employed doctors who were born in Africa and have lived long enough in the destination country to be part of that country's census.

^b^Emigration fraction: the ratio of the number of physician émigrés to the sum of the number of physicians remaining at home in Africa and the number of émigrés.

^c^Physician migration density: the number of physician émigrés per 1000 population of each African country.

**Figure 1:**
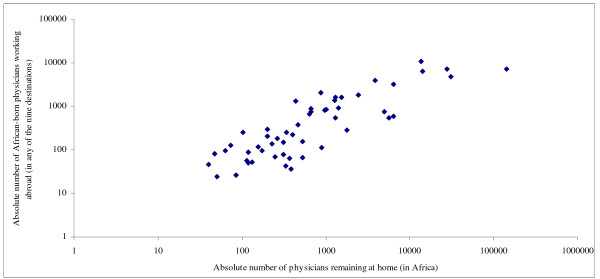
Correlation between number of physicians working abroad and number of physicians remaining at home.

**Figure 2:**
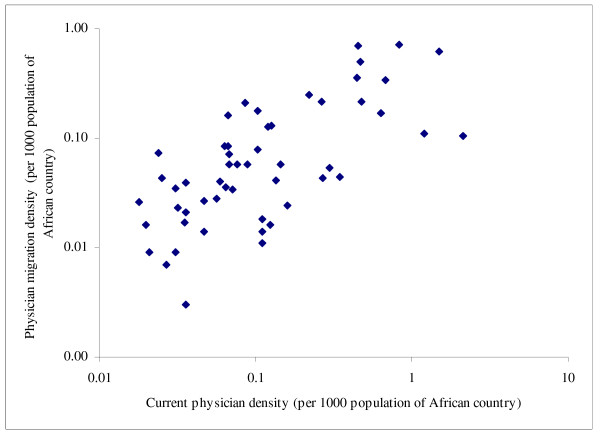
Correlation between physician migration density and current physician density (log-transformed).

**Figure 3:**
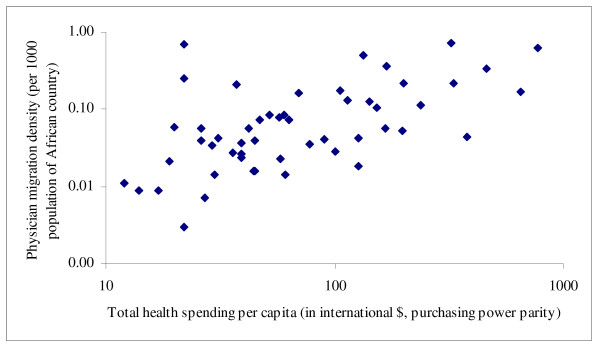
Correlation between physician migration density and total health spending per capita (log-transformed).

**Figure 4:**
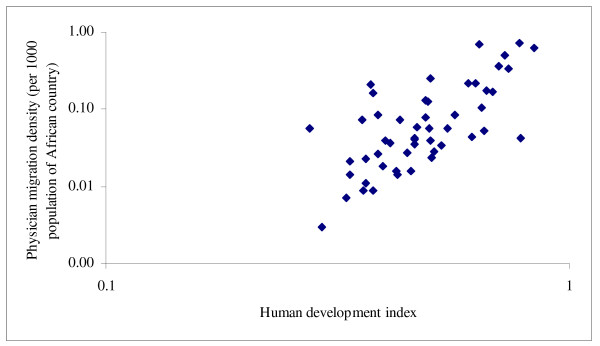
Correlation between physician migration density and human development index (log-transformed).

Table 2 shows that the absolute number of émigrés had few associations with source countries' characteristics. It only correlated positively with physician density, healthy life expectancy and female literacy but negatively with poverty and share of total spending from external resources. Emigration fraction showed no discernible cross-national patterns except for its expected correlation with current physician density (considering that the denominator of the emigration fraction and the numerator of the physician density both contained the number of physicians remaining in each African country). The physician migration density, however, showed consistent and significant associations with source countries' characteristics: higher physician emigration appeared to occur among African countries with relatively higher health workforce capacity (0.401 ≤ *r *≤ 0.694, *p *≤ 0.011), better health status, higher health system spending (*r = *0.583, *p *< 0.001) but lower official development assistance for health (*r *= -0.385, *p *= 0.005), and better economic and social development. Figures 1, 2, 3, 4 show selected scatter plots of these significant correlations.

**Table 2:** Correlates of physician migration from African countries

**Variable**	**Total number of African physician émigrés**	***p *value**	**Emigration fraction**	***p *value**	**Physician migration density**	***p *value**
**Health-workforce**						
Current physician density	0.353	0.010	-0.352	0.010	0.694	<0.001
Current nurse density	0.222	0.111	-0.173	0.216	0.601	<0.001
Medical school density	-0.086	0.609	-0.106	0.527	0.401	0.011
						
**Health status**						
Infant mortality rate	-0.213	0.125	0.152	0.279	-0.654	<0.001
Under-five mortality rate	-0.247	0.074	0.136	0.333	-0.676	<0.001
Healthy life expectancy at birth	0.285	0.039	-0.127	0.366	0.632	<0.001
						
**Health system spending**						
Total health spending	0.189	0.180	-0.072	0.612	0.583	<0.001
Share of health spending from external resources	-0.461	0.001	0.289	0.038	-0.385	0.005
						
**Economic and social development**						
Gross national income per capita	0.140	0.352	-0.144	0.338	0.534	<0.001
Poverty	-0.477	0.006	0.244	0.178	-0.472	0.006
Female literacy	0.298	0.047	0.280	0.062	0.576	<0.001
Human development index	0.180	0.207	0.063	0.663	0.703	<0.001

## Discussion

Rankings of physician migration based on the number of physician émigrés from African, emigration fraction and physician density produce different results. Only the physician migration density and, to a lesser extent, the number of physician émigrés show a systematic pattern of associations with health workforce, health status, health spending and social-economic profiles of African countries. This study highlights an important but often neglected problem in studies and reports which quantify the magnitude and patterns of health workforce migration: metrics tell tales and quite often different ones, depending on the perspectives adopted.

Although this study is limited by its focus on one continental – African – experience, as far as the author knows, it is novel in looking at the impact of choice of migration metrics on the perception of which source countries may suffer relatively more physician emigration. Like other health workforce studies, this study is also limited by its use of metrics which quantify stock, rather than actual flow over time [22,23]. Unfortunately, few studies can reliably have the luxury of data on time-dependent flow of health-workers. That said, this study is among the first to use the new database which accounts for bilateral net flows among source and other countries. The quality of these data is not necessarily comparable or completely reliable across countries although it has been improving over the last few years [1,4,13,17,20]. Differential bias in the results could occur if the quality of the health workforce and migration data is shown to be systematically associated with the observed levels of the countries' profiles. Partly based on how well these data have worked in global health analyses and the intuitive nature of the results, I have little reason to suspect any substantial or differential bias in the findings which could arise from the varying data quality.

Other limitations are given in Clemens and Pettersson [13], although their data which were used here tried to overcome some of the standard problems of existing health workforce data such as focusing only on physicians trained in own birth country. As discussed elsewhere, using birth country to classify physicians may reflect the extent of "Africa-ness" of the physicians although this need not be suitable for every health workforce research [13]. Considering my experience with a recent analysis of a different global database of more than one hundred and forty countries which lost physicians to the US, Canada, Australia and UK [2] and in which the physician migration metric was originally proposed [20], classifying the physician émigré according to country of medical training yields similar migration correlates as the current study. As one of the reviewers of the current study thoughtfully pointed out, using only African-born physicians in the denominator of the emigration fraction might overstate the magnitude of migration or yield misleading results because foreign-born physicians who remained active in Africa would not be counted. Including foreign-born physicians in the denominator of the emigration fraction, however, also implies that they should be included in the numerator whenever they emigrate. Otherwise, the emigration fraction might paint the wrong picture since the foreign-born physicians would – inappropriately and – statistically 'not be allowed to be at risk' of emigration.

Given its scope and ecological design, this paper does not and cannot address the correlates of why individual physicians emigrate. For such analysis, researchers would need coupled hierarchical data, nesting individual physician émigrés within both destination and source countries, to avoid cross-level inferential fallacies [20]. Furthermore, this study does not pretend to answer the question of who an African physician émigré should be [13]. Is it a doctor born in Africa? Or is it a doctor who just holds an African citizenship or a physician trained in Africa? This study made use of a database which classified the African physician as someone born in Africa, currently employed as a medical doctor, and had been residing in the destination country long enough to be included in the country's recent census [13]. This definitional choice does not detract from the central thrust of this study which is to show how the extent and patterns of migration might be dependent on the type of metric used.

Unlike other studies which have also addressed the African migration crisis [1-3,13,24], this paper emphasizes that, although the emigration fraction is useful for indicating the extent of workforce losses through migration, it is not designed to account for the importance of the population size or to pattern migration according to national contextual profiles of the source countries [20]. By relating to the size of the physician pool, the emigration fraction intuitively outperforms the total number of émigrés metric. Nonetheless, the emigration fraction differs from the physician migration density which adjusts for source population size in its ability to depict the macro-patterns of migration. At first glance, the correlates of migration might seem counterintuitive [20], but a closer look reveals that somewhat richer African countries like Seychelles (1.51), Mauritius (1.06) and Tunisia (1.34) also have higher physician densities per 1000 population than the average African country (0.27). Also, higher physician capacity and wealth are usually seen in countries with higher health spending, less poverty and better overall development [1,4,19,20,23]. It is, therefore, not surprising that physician migration density is also positively associated with development-related profiles. Previous studies have tended to allude qualitatively to the poorer profiles of countries with higher emigration fractions. This study goes further and assesses the actual correlations and finds that the emigration fraction was not patterned according to common national profiles. Like Mejia's landmark study in 1978 [25], this paper shows that migration has a positive gradient with source countries' capacity [20,21]. This study suggests that the emigration fraction may be more appropriate for depicting physician stock depletion while the migration density is more appropriate for understanding country-level patterns in emigration [20]. Recent analysis reveals that the methods used in this study work well on nurse migration data and yield similar findings [26].

So, what do these findings mean for policy and future research? Policies [8,11,27] being suggested for solving the migration threats to the health workforce in Africa and other poor areas might be barking up the wrong tree [28]. If wealthier North American and European countries draw relatively more physicians from less poor countries with which they may have better visa prospects, recognition of educational qualifications, and foreign relations [20,29], in the long-term, it is possible that migration reversal and retention policies might benefit the 'rich' but not necessarily the very poor source countries which have absolutely and relatively insufficient physicians to begin with [20]. This does not imply that every physician who returned to a physician-poor setting would not improve the supply of that country. Treating more patients could make a big difference to the suffering patients and their families but the impact would be hard to gauge at the population level in countries with very low physician densities but high disease burden [20]. Unfortunately, this scenario is not far-fetched in many African countries. Policymakers need to be careful about seeing migration reversal as a long-term strategic solution to health workforce shortages.

## Conclusion

Policymakers and researchers must begin to look into the conceptual, methodological and interpretational issues surrounding migration metrics while considering the causes, consequences, and solutions of health-worker migration. Given the challenges faced by Africa and the centrality of the health workforce in achieving the Millennium Development Goals or any health goals [21], all issues surrounding her workforce demand critical analysis and enduring commitment.

## Competing interests

The author(s) declare that they have no competing interests.

## Authors' contributions

OAA is the sole author of this paper.

## Pre-publication history

The pre-publication history for this paper can be accessed here:



## Supplementary Material

Additional File 1 **All 53 African countries included in the study**. (File in Microsoft^® ^Word format; a list of all 53 African countries included in the study) Click here for file

Additional File 2 **Definitions, data sources, and descriptive statistics of variables used in the study**. (File in Microsoft^® ^Word format; extensive definitions, data sources and descriptive summary statistics of all variables included in the study) Click here for file

Additional File 3 **Top 10 and bottom 10 African countries which suffer the most and least physician migration respectively, ranked by 3 different migration metrics – total number of physician émigrés, emigration fraction, and physician migration density**. (File in Microsoft^® ^Word format; list of top 10 and bottom 10 African countries that suffer the most and least physician emigration respectively, ranked by 3 different migration metrics) Click here for file
