# Supplementary material for: The metrics and correlates of physician migration from Africa
Source: BMC Public Health. 2007 May 17;7:83. doi: 10.1186/1471-2458-7-83 (PMC1885251; doi:10.1186/1471-2458-7-83)
Supplement: Additional File 1 — All 53 African countries included in the study. (File in Microsoft® Word format; a list of all 53 African countries included in the study) [file 1471-2458-7-83-S1.doc]

**Additional file 1: All 53 African countries included in the study**

Algeria

Angola

Benin

Botswana

Burkina Faso

Burundi

Cameroon

Cape Verde

Central African Republic

Chad

Comoros

Congo

Côte d'Ivoire

Democratic Republic of the Congo

Djibouti

Egypt

Equatorial Guinea

Eritrea

Ethiopia

Gabon

Gambia

Ghana

Guinea Bissau

Guinea

Kenya

Lesotho

Liberia

Libyan Arab Jamahiriya

Madagascar

Malawi

Mali

Mauritania

Mauritius

Morocco

Mozambique

Namibia

Niger

Nigeria

Rwanda

São Tomé and Principe

Senegal

Seychelles

Sierra Leone

Somalia

South Africa

Sudan

Swaziland

Togo

Tunisia

Uganda

United Republic of Tanzania

Zambia

Zimbabwe
