# Supplementary material for: The metrics and correlates of physician migration from Africa
Source: BMC Public Health. 2007 May 17;7:83. doi: 10.1186/1471-2458-7-83 (PMC1885251; doi:10.1186/1471-2458-7-83)
Supplement: Additional File 2 — Definitions, data sources, and descriptive statistics of variables used in the study. (File in Microsoft® Word format; extensive definitions, data sources and descriptive summary statistics of all variables included in the study) [file 1471-2458-7-83-S2.doc]

**Additional file 2: Definitions, data sources, and descriptive statistics of variables used in the study**

| **Variable**  **Definition** | | **Data source (data year)** | **Mean (standard deviation) among source countries in Africa** | **Mean (standard deviation) among the 9 destination countries** |
| --- | --- | --- | --- | --- |
| **Health-workforce** | |  |  |  |
| Current physician density | Number of physicians per 1000 population | WHO ^w1^ (1995-2004) and CGD^w2^ | 0.24 (0.39) | 2.76 (1.05) |
| Current nurse density | Number of nurses per 1000 population | WHO^w1^ (1997-2004) | 1.35 (1.61) | 7.82 (2.72) |
| Medical school density | Number of medical schools per million population | JLI^w3^ and WHO^w4^ (2000) | 0.21 (0.20) | 0.57 (0.24) |
| **Health status** | |  |  |  |
| Infant mortality rate | Number of infants dying before reaching age one year, per 1000 live births in a given year | WHO ^w5^  (2000) | 87.82 (37.39) | 10.22 (14.55) |
| Under-five mortality rate | Probability (expressed as a rate per 1,000 live births) of a child born in a specified year dying before reaching the age of five if subject to current age-specific mortality rates | WHO ^w5^  (2000) | 142.40 (63.31) | 13.70 (21.59) |
| Healthy life expectancy (HALE) at birth | Life expectancy at birth in a given country, taking into account time spent in poor health | WHO^w5^  (2000) | 41.76 (8.50) | 66.53 (8.91) |
| **Health system spending** | |  |  |  |
| Total health spending | Total expenditure on health per capita, measured in international dollars (based on purchasing power parity) | JLI^w3^ and WHO^w6^ (2001) | 119.48 (155.50) | 2,347 (1163) |
| Share of health spending from external resources | Percentage of total expenditure on health from external resources (official development assistance for health) | JLI^w3^ and World Bank ^w7^ (2001) | 20.34 (16.83) | -^##^ |
| **Economic and social development** | |  |  |  |
| Gross national income per capita | Gross national income (wealth) per capita, measure in international dollars (based on purchasing power parity) | JLI^w3^ and World Bank^w7^ (2001) | 2,475 (2,519) | 23,165 (6,509) |
| Poverty | Percentage of the population living on less than one international dollar (based on purchasing power parity) per day | JLI^w3^ and World Bank ^w7^ (1993-2001) | 38.69 (25.08) | -^##^ |
| Female literacy | Percentage of female adults above 15 years who are literate | World Bank ^w7^ (2001) | 53.47 (21.53) | 90.67 (6.03) |
| Human development index | A composite index that measures the average achievements in a country in three basic dimensions of human development: a long and healthy life, as measured by life expectancy at birth; knowledge, as measured by the adult literacy rate and the combined gross enrolment ratio for primary, secondary and tertiary schools; and a decent standard of living, as measured by GDP per capita in purchasing power parity (PPP) US dollars | United Nations^w8^ (2000) | 0.50 (0.14) | 0.90 (0.08) |

^#^United Kingdom, United States, France, Canada, Australia, Belgium, Portugal, Spain, and South Africa

^##^- value close to zero due to too many missing values

**References for additional file 2:**

^w1^ World Health Organization (WHO). *World Health Report 2006. Working Together for Health*. (Statistical annexes.) Geneva: WHO, 2006.

^w2^ Clemens MA, Pettersson G. *A New Database of Health Professional Emigration from Africa*. Washington, D.C.: Center for Global Development (CGD), 2006.

^w3^ Joint Learning Initiative (JLI). *Human Resources for Health: Overcoming the Crisis*. Cambridge, MA: The President and Fellows of Harvard College, 2004.

^w4^ World Health Organization (WHO). *World Directory of Medical Schools*. 7th Edition. Geneva: WHO, 2000.

^w5^ World Health Organization (WHO). *World Health Report 2001.* (Statistical annexes.) Geneva: WHO, 2001.

^w6^ World Health Organization (WHO). *World Health Report 2004.* (Statistical annexes.) Geneva: WHO, 2004.

^w7^ World Bank. *World Development Indicators 2005.* CD-ROM. Washington, D.C.: The World Bank, 2005.

^w8^ United Nations Development Programme (UNDP). *Human Development Report Data.* <http://hdr.undp.org/statistics/data/> (accessed January 13, 2006).
