# Supplementary material for: The metrics and correlates of physician migration from Africa
Source: BMC Public Health. 2007 May 17;7:83. doi: 10.1186/1471-2458-7-83 (PMC1885251; doi:10.1186/1471-2458-7-83)
Supplement: Additional File 3 — Top 10 and bottom 10 African countries which suffer the most and least physician migration respectively, ranked by 3 different migration metrics – total number of physician émigrés, emigration fraction, and physician migration density. (File in Microsoft® Word format; list of top 10 and bottom 10 African countries that suffer the most and least physician emigration respectively, ranked by 3 different migration metrics) [file 1471-2458-7-83-S3.doc]

**Additional file 3: Top 10 and bottom 10 African countries which suffer the most and least physician migration respectively, ranked by 3 different migration metrics—total number of physician émigrés, emigration fraction, and physician migration density**

| ***Top 10 source countries*** | | |
| --- | --- | --- |
| **Number of physician émigrés** | **Emigration fraction** | **Physician migration density** |
| 1. Algeria | 1. Mozambique | 1. Mauritius |
| 1. South Africa | 1. Guinea-Bissau | 1. São Tomé and Principe |
| 1. Egypt | 1. Angola | 1. Seychelles |
| 1. Morocco | 1. Liberia | 1. Cape Verde |
| 1. Nigeria | 1. Equatorial Guinea | 1. Algeria |
| 1. Kenya | 1. São Tomé and Principe | 1. Tunisia |
| 1. Tunisia | 1. Malawi | 1. Congo |
| 1. Angola | 1. Zambia | 1. Morocco |
| 1. Uganda | 1. Ghana | 1. Namibia |
| 1. Ghana | 1. Congo | 1. Guinea-Bissau |
|  |  |  |
| ***Bottom 10 source countries*** | | |
| 1. Botswana | 1. Cote d'Ivoire | 1. Somalia |
| 1. Gabon | 1. Sudan | 1. Rwanda |
| 1. Lesotho | 1. Guinea | 1. Mauritania |
| 1. Swaziland | 1. Botswana | 1. Mali |
| 1. Seychelles | 1. Mauritania | 1. Guinea |
| 1. Gambia | 1. Democratic Republic of the Congo | 1. Democratic Republic of the Congo |
| 1. Mauritania | 1. Niger | 1. Ethiopia |
| 1. Niger | 1. Libyan Arab Jamahiriya | 1. Chad |
| 1. Djibouti | 1. Egypt | 1. Burkina Faso |
| 1. Comoros | 1. Cote d'Ivoire | 1. Niger |
